# Enterobacterales abundance in oral cancer patients and elevated clindamycin resistance rates in head and neck infections at a Hungarian Tertiary Hospital

**DOI:** 10.1186/s12941-025-00802-x

**Published:** 2025-05-29

**Authors:** Dorottya Diana Kiss, Zsolt Nemeth, Daniel Sandor Veres, Krisztina Marton, Arpad Joob-Fancsaly, Katalin Kristof

**Affiliations:** 1 Department of Oro-Maxillofacial Surgery and Stomatology, Semmelweis University, Budapest, 1085 Hungary; 2 Department of Biophysics and Radiation Biology, Semmelweis University, Budapest, 1094 Hungary; 3 Department of Preclinical Dentistry, Semmelweis University, Budapest, 1088 Hungary; 4 Department of Laboratory Medicine, Semmelweis University, Budapest, 1083 Hungary

**Keywords:** Oral microbiology, Antimicrobial resistance, Oral cancer, Bacterial pathogens, Head and neck infections, Clindamycin

## Abstract

**Background:**

Oral bacteria have been associated with several systemic diseases, and studies have highlighted their potential role in carcinogenesis. A biofilm is considered an antimicrobial resistance gene reservoir, and the oral cavity provides an excellent environment for biofilm formation. The aim of this study was to evaluate the pathogen spectrum and antimicrobial resistance rates of clinical isolates from head and neck infections in the Hungarian population.

**Methods:**

A total of 5185 bacterial isolates were analyzed from 1978 patients between 2018 and 2023. Antimicrobial resistance rates were reported according to the EUCAST guidelines. The primary diagnoses of the patients were categorized into three major groups: abscesses, necrotizing lesions and surgical site infections of patients treated for malignant tumors. Pearson’s chi-square test was used to compare the percentages of bacteria in the different patient groups.

**Results:**

The most frequently isolated bacteria were *Streptococcus* (18.8%) and *Prevotella* spp. (13.5%), followed by *Staphylococcus* (13.2%) and *Fusobacterium* spp. (9.1%). Differences in the pathogen spectrum of three patient groups (‘abscess’, ‘necrosis’ and ‘tumor’) were also evaluated. Compared with the other two patient groups, cancer patients had significantly greater percentages of *Enterobacter* spp., *Enterococcus* spp., *Pseudomonas* spp. and beta-hemolytic streptococci. Substantial resistance rates to clindamycin were observed for *Prevotella*, *Streptococcus* and *Staphylococcus* spp. at 40.9% (95% CI [37.3–44.7%]), 34.8% (95% CI [31.8–37.9%]) and 32.3% (95% CI [28.8–35.9%]), respectively. The percentage of methicillin-resistant *Staphylococcus aureus* isolates was 13.8% (95% CI [9.2–19.5%]). The percentage of vancomycin-resistant *Enterococcus* spp. isolates was 2.8% (95% CI [0.6–8.0%]), and the percentages of extended-spectrum beta-lactamase-producing *E. coli* and *Klebsiella* spp. isolates were 1% (95% CI [0.02–5.6%]) and 2.6% (95% CI [0.8–5.9%]), respectively.

**Conclusion:**

Our evaluation revealed high percentages of Enterobacterales in patients with diseases such as osteonecrosis or oral cancer. Further investigation of the role of the oral microbiota and its potential impact on the morbidity of patients with advanced disease is needed. Substantial antimicrobial resistance rates, particularly to clindamycin, pose a major concern for treating bacterial infections in the head and neck region.

**Supplementary Information:**

The online version contains supplementary material available at 10.1186/s12941-025-00802-x.

## Background

Antimicrobial resistance (AMR) has become one of the most concerning global health threats. According to a systematic analysis evaluating the global burden of bacterial AMR, in 2019, an estimated 4.95 million deaths were caused by drug-resistant infections, of which 1.27 million deaths were attributable to bacterial AMR [1]. A forecast based on the findings of a systematic analysis predicted a continuous increase in mortality related to AMR, with an estimated 1.91 million deaths attributable to AMR and 8.22 million deaths associated with AMR by 2050 [2]. Six pathogens (*Escherichia coli*, *Staphylococcus aureus*, *Klebsiella pneumoniae*, *Streptococcus pneumoniae*, *Acinetobacter baumannii*, and *Pseudomonas aeruginosa*) have each been found to be responsible for more than 250,000 deaths, and six more pathogens (*Mycobacterium tuberculosis*, *Enterococcus faecium*, *Enterobacter* spp., *Streptococcus agalactiae*/group B streptococci, *Salmonella typhi*, and *Enterococcus faecalis*) have each been found to be responsible for more than 100,000 deaths associated with AMR [1]. Although most of these bacteria are not predominant pathogens of the maxillofacial region, in certain cases, the bacterial flora can be overturned, and the abovementioned species can become major pathogens of head and neck infections. Before the widespread use of semisynthetic penicillin derivatives, most odontogenic infections were known to be streptococcal. This selective genetic pressure then resulted in infections from enteric and opportunistic bacteria (e.g., vancomycin-resistant enterococci and methicillin-resistant staphylococci) through mutation and gene transfer. Changes in the normal flora can also be induced by antibiotics or the use of immunosuppressive drugs, and certain conditions, such as hospitalization, can result in the acquisition of new flora [3].

Several factors can contribute to the spread of AMR. The major drivers of AMR spread are the overuse and inappropriate use of antimicrobial agents [1]. According to the World Health Organization (WHO) and the U.S. Centers for Disease Control and Prevention (CDC), the misuse of antibiotics during the coronavirus disease 2019 (COVID-19) pandemic could have accelerated the development of drug-resistant pathogens [4–6].

Bacteria have developed various strategies to counteract the effects of antibiotics, such as decreasing drug uptake, enhancing efflux mechanisms, modifying antibiotic targets or enzymes, and altering metabolic pathways. The dissemination of resistance is primarily facilitated by the transfer of antibiotic resistance genes through mobile genetic elements, including plasmids, transposons, and integrons. Clindamycin resistance is primarily mediated through ribosomal methylation by erm genes, which modify the 23S rRNA and prevent the binding of clindamycin and macrolides, leading to resistance. Additional mechanisms include efflux pumps, target site mutations, and horizontal gene transfer, with inducible resistance often triggered by macrolides, complicating treatment [7, 8].

Although global monitoring of the spread of AMR is based on reports of bloodstream infections [4, 9], identifying pathogens at the site of infection and their resistance rates is also fundamental from a clinical perspective. This approach is particularly relevant because the oral cavity provides an excellent environment for biofilm formation on teeth and foreign bodies (e.g., dental materials, medical devices, titanium implants or reconstruction plates).

The oral microbial community, with approximately 1000 species, is the second largest microbiota in the human microbiome after the colon. Biofilm formation can have severe consequences for patients since microorganisms in a biofilm are less susceptible to antibiotics and host defenses than those in a planktonic form, and the biofilm itself is also considered an AMR gene reservoir. It is widely accepted that there is a connection between various systemic diseases and oral microbial pathogens, which can affect the gastrointestinal and cardiovascular systems and can also have an effect on diabetes, premature labor, arthritis and mental illness. Biofilm infections can also account for clinical challenges such as impaired wound healing or diseases caused by unculturable bacteria, and biofilm infections can also lead to rapid development of AMR or the spread of infectious emboli [10–13]. Engineered bacteriophages have a potential to disrupt biofilms and overcome traditional antimicrobial resistance mechanisms which offers a promising therapeutic potential against multidrug-resistant bacteria [14].

Several studies have demonstrated differences between the microbiota at tumorous and healthy sites in oral cancer patients, highlighting the potential role of highly prevalent biofilm-associated dental diseases and chronic inflammation in carcinogenesis, particularly since poor oral hygiene is common among these patients [15–20]. Smoking and alcohol consumption may also contribute to changes in the composition of the oral microbiota. The resident microflora has beneficial protective properties, and an overrepresentation of pathogenic bacteria might play a role in carcinogenesis by inducing inflammatory responses. Bacterial flagella are considered key structures in the regulation of this process. Moreover, genetic alterations in the epithelial cells of the host might be induced by bacterial products and metabolic byproducts [18, 21, 22]. This study is the first extensive microbiological evaluation of maxillofacial infections in Hungary. The aim of our study was to evaluate the spectrum of clinical pathogens and the AMR rate of patients treated for infections of the head and neck region in a tertiary-care hospital. We compared these data with the primary diagnoses of the patients who were treated at our clinic to assess the correlation between them. To our knowledge, this study is the first to directly compare the pathogen spectrum of patients with oral cancer to that of patients with benign oral lesions, specifically abscesses and necrosis. Previous research has focused primarily on either cancerous tissues or benign lesions separately, but no comprehensive comparison of the pathogen spectrum between these groups has been conducted. Finally, we compared our results with data from other countries. Because the Hungarian population generally has poor oral hygiene [23], it is important to assess the most common pathogens and compare them with international data to implement a greatly needed antibiotic stewardship program for dentists and oral surgeons.

## Materials and methods

A retrospective analysis was conducted on the clinical isolates of patients who had been treated for infections of the head and neck region at the Department of Oro-maxillofacial Surgery and Stomatology (Semmelweis University) between 2018 and 2023. Patients from both inpatient and outpatient care who had samples taken during their treatment were included in the study. No patients were excluded based on clinical characteristics or other factors. Samples were taken from a variety of maxillofacial infections, such as abscesses, osteonecrosis sites or postoperative surgical site infections (SSIs). In the case of odontogenic infections, samples were taken after an incision was made from the cavity of the abscess. A large portion of the isolates were derived from patients who developed an SSI after an operation for a malignant tumor. The exudates were sampled either intraorally or extraorally, depending on the location of the infection. Samples were taken with swabs and delivered to the Microbiology Laboratory of the Department of Laboratory Medicine (DLM) in transport medium (Amyes transport swabs), where microbiological culturing and antimicrobial susceptibility testing were performed. After aerobic and anaerobic cultivation, the bacteria were identified by a matrix-assisted laser desorption ionization–time of flight mass spectrometry (MALDI-TOF MS) system (Bruker Ltd., USA). Antimicrobial susceptibility testing was performed, and the results were evaluated according to the current European Committee on Antimicrobial Susceptibility Testing (EUCAST) recommendations [24]. The analysis of data was based on a review of patient records in the university electronic medical system (age, sex and primary diagnosis). Patients were categorized according to their diagnoses and for further analysis, they were identified with code numbers to protect personal data. For the handling of patient data, Regulation 2016/679 of the European Parliament and of the Council on General Data Protection was implemented.

In terms of the total number of bacteria, only those that could be detected at least 10 times per year and were considerable pathogens throughout the 6-year period were included in the final evaluation of the bacterial spectrum and AMR pattern. Isolates that recurred in the same patient within 3 months were excluded if there were no changes in antimicrobial susceptibility. For the sake of comprehensibility, certain bacteria that have similar characteristics were grouped together; for example, *Citrobacter*, *Morganella*, *Proteus*, *Serratia* spp. and *Klebsiella aerogenes* were included in the *Enterobacter* spp. group, *Porphyromonas* spp. were included in the *Prevotella* spp. group, and *Alloscardovia* and *Dialister* spp. were included in the *Veillonella* spp. group. *Streptococcus* spp. were classified as beta-hemolytic and ‘other’ streptococci, the latter essentially consisting of viridans group streptococci. This classification is in accordance with the EUCAST suggestions. *Staphylococcus* spp. were categorized into *S. aureus* and ‘other’ staphylococci*,* which included basically coagulase-negative staphylococci. We reported the rates of resistance only for those antibiotics that were recommended by the EUCAST guidelines. The AMR rates are expressed as percentages with 95% confidence intervals (CIs). To compare the percentages of bacteria in the different patient groups, Pearson’s chi-square test was conducted with IBM SPSS Statistics 30. The significance level was set at α = 0.05. For the chi-square test, recurring bacterial isolates were completely excluded, and the bacteria were evaluated by genera instead of species to reduce bias. For the descriptive analysis, patient sex was assigned as female or male, and patients were categorized into age groups (0–19, 20–39, 40–59, 60–79, and 80–99 years). The study was approved by the Regional, Institutional Scientific and Research Ethics Committee of Semmelweis University (date of approval: 18 January 2024, approval number: 270/2023), and participants provided written informed consent.

The outcomes of this study were as follows: the pathogen spectrum of oral and maxillofacial infections in a tertiary-care hospital in Hungary, a comparison of the percentage distribution of bacteria in different patient groups and the AMR rates of the twelve most common pathogens.

## Results

A total of 5654 species were isolated between 2018 and 2023. After the exclusion of recurring bacteria in one period of infection, 5185 clinical isolates from 1978 patients were ultimately included in this study. The ages of the patients ranged from 4 to 94 years, and most samples (68%) were taken from patients older than 40 years of age. The male-to-female ratio was 1.125. The number of isolates increased more than twofold from 2018 to 2023 (Table 1).

**Table 1:** Baseline characteristics of patients treated for head and neck infections at the Department of Oro-Maxillofacial Surgery and Stomatology (Semmelweis University, Budapest) and the year of sampling

	n	%
Patient		
Sex		
Female	931	47.1
Male	1047	52.9
Age, years		
0–19	97	4.90
20–39	536	27.10
40–59	529	26.74
60–79	683	34.53
80–99	133	6.72
Isolate		
Year of sampling		
2018	434	8.4
2019	880	17.0
2020	813	15.7
2021	998	19.2
2022	991	19.1
2023	1069	20.6

### Overall pathogen spectrum of head and neck infections

**Fig. 1:**
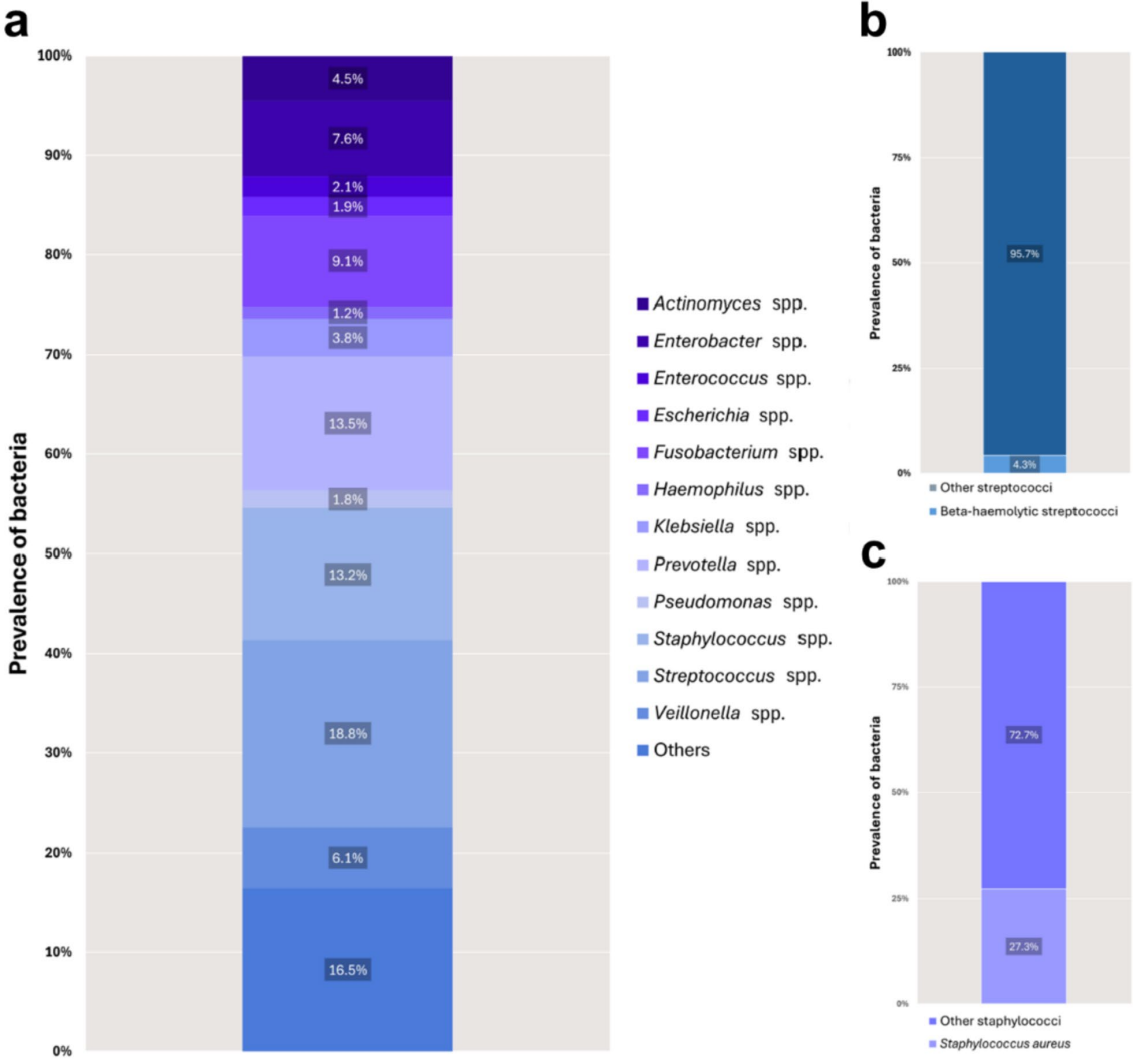
Percentage distribution of bacteria identified from infections of the head and neck region. **a** Overall bacterial spectrum (n = 5185). **b** Percentages of beta-hemolytic (n = 39) and ‘other’ streptococci (n = 935) isolates. **c** Percentages of *S. aureus* (n = 189) and ‘other’ staphylococci (n = 495) isolates

**Fig. 2:**
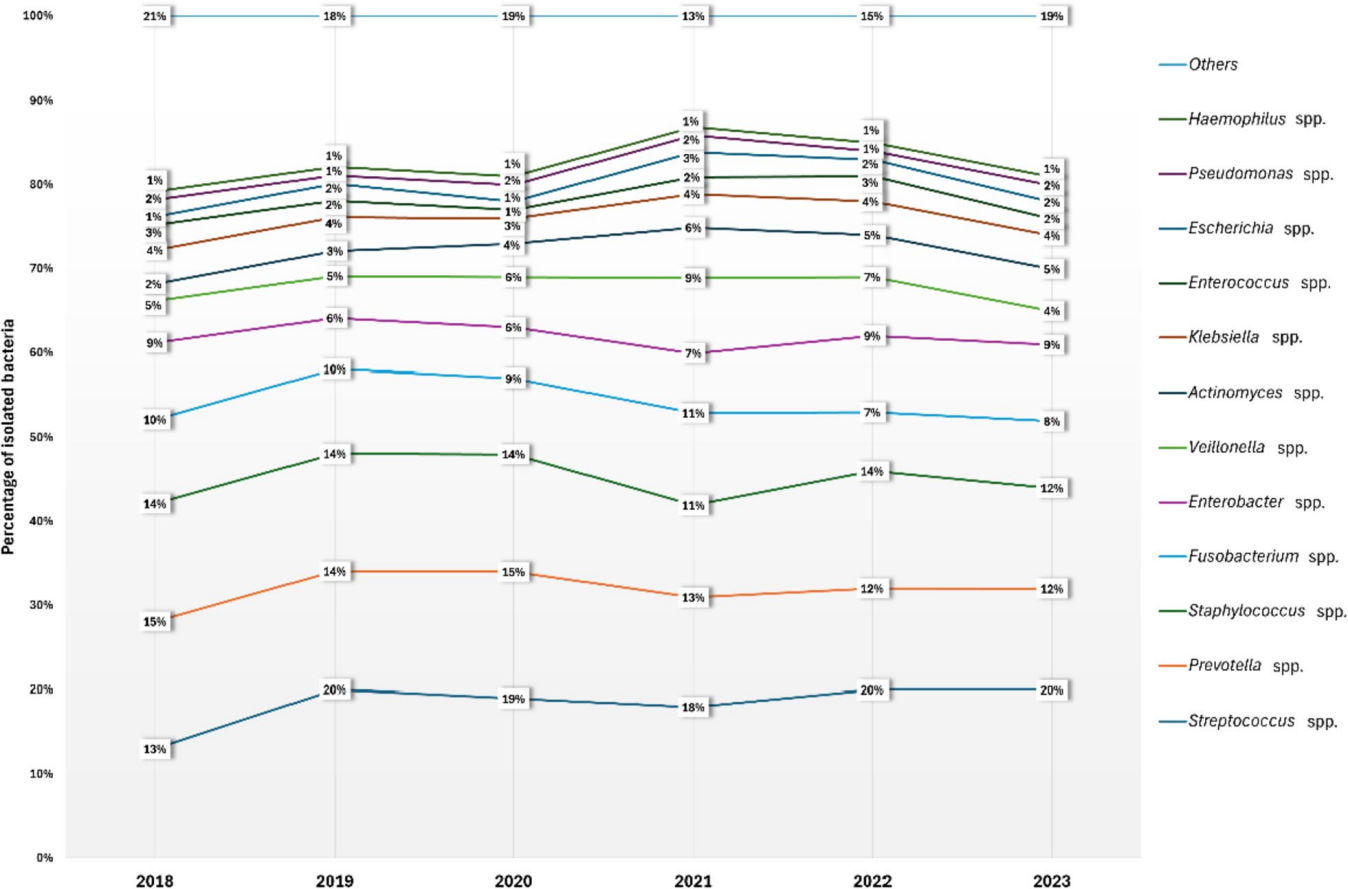
Annual proportional distribution of bacteria from clinical isolates of patients treated for head and neck infections throughout the study period (2018–2023)

Through bacterial cultures, a total of 290 different species from 88 genera were identified. The prevalence of the most relevant bacteria for the entire population is shown in Fig. 1 and Additional file 1: Table 1. The most prevalent bacteria were *Streptococcus* spp. (18.8%), *Prevotella* spp. (13.5%), *Staphylococcus* spp. (13.2%) and *Fusobacterium* spp. (9.1%). Beta-hemolytic streptococci accounted for 4.0% of all streptococci*,* and *S. aureus* accounted for 27.6% of all staphylococci. Notably, 60.5% of the staphylococcal isolates consisted of *Staphylococcus epidermidis*. A substantial number of *Enterobacter* spp. (7.6%) were also identified. The prevalences of the different bacteria did not considerably change over the years (Fig. 2).

### Spectrum of pathogens in the different patient groups

The primary diagnoses of the patients could be categorized into three major groups: abscesses (mainly odontogenic infections, n = 900), necrotizing lesions (mainly osteonecrosis, n = 332) and surgical site infections of patients treated for malignant tumors [particularly oral squamous cell carcinoma (OSCC), n = 179]. We found this categorization worthwhile since the distribution of bacteria differed substantially in each group. In this instance, the less prevalent bacteria (previously assigned as ‘others’) were not included in the calculations for clarity, resulting in 4327 isolates from 1411 patients for the analysis.

**Fig. 3:**
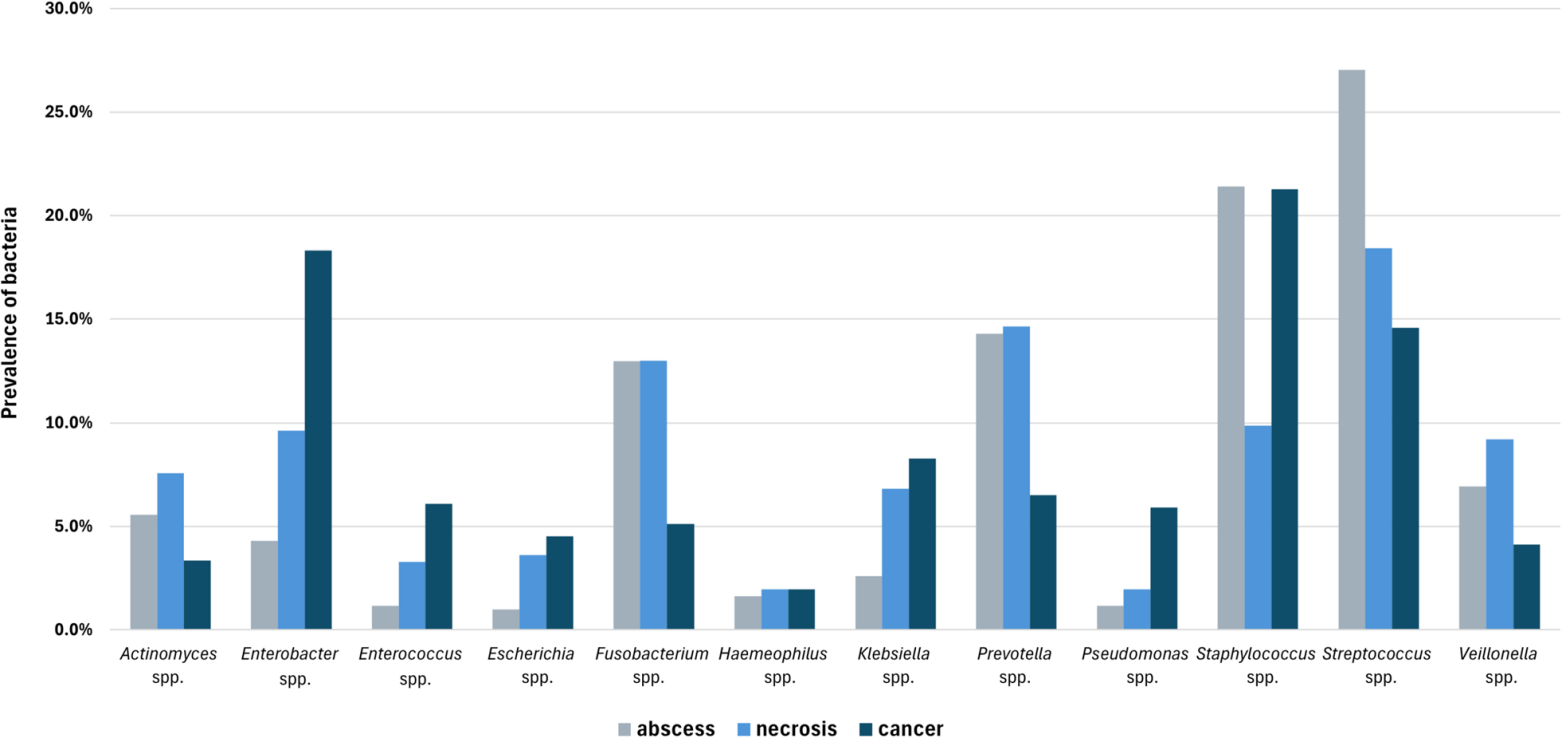
Percentages of bacteria in the different patient groups

The percentages of pathogens in the different patient groups are shown in Fig. 3. The results after the exclusion of recurring bacterial isolates are provided in Additional file 1: Table 2. Despite the exclusion of some isolates, there were no essential changes in the percentages (Additional file 1: Fig. 1), other than the fact that instead of *Staphylococcus spp*., *Enterobacter* spp. were originally the most frequently isolated bacteria in patients with malignancies.

A comparison of the pathogen spectra of the different groups revealed that patients with abscesses and osteonecrosis had some similarities, but significantly greater percentages of *Enterobacter* spp. (4.3% vs. 9.6%), *Actinomyces* spp. (5.6% vs. 7.6%), *Klebsiella* spp. (2.6% vs. 6.8%), *Enterococcus* spp. (1.2% vs. 3.3%) and *Escherichia* spp. (1.0% vs. 3.6%) and significantly lower percentages of *Streptococcus* spp. (27.0% vs. 18.4%) and *Staphylococcus* spp. (21.4% vs. 9.9%) (p < 0.05, Table 2) were detected in the ‘necrosis’ group. Among patients who were treated for oral cancer, *Enterobacter* spp. were the second most common pathogen (18.3%), after staphylococci (21.3%). This group also had significantly higher percentages of *Pseudomonas* spp. (5.9%), *Enterococcus* spp. (6.1%) and beta-hemolytic streptococci (2%, accounting for 13.5% of all streptococci) and significantly lower percentages of *Fusobacterium* spp. (5.1%) than the other two groups (p < 0.05).

**Table 2:** Comparison of the percentages of bacteria in the different patient groups (df = 1, p < 0.05). Significant results are marked in bold, and the groups that had significantly higher percentages are indicated in parentheses

	Patient groups compared		
	Abscess—necrosis	Abscess—tumor	Necrosis—tumor
	*p* value		
*Actinomyces* spp.	**0.028** (necrosis)	**0.046** (abscess)	**0.001** (necrosis)
*Enterobacter* spp.	**< 0.001** (necrosis)	**< 0.001** (tumor)	**< 0.001** (tumor)
*Enterococcus* spp.	**< 0.001** (necrosis)	**< 0.001** (tumor)	**0.007** (tumor)
*Escherichia* spp.	**< 0.001** (necrosis)	**< 0.001** (tumor)	0.373
*Fusobacterium* spp.	0.981	**< 0.001** (abscess)	**< 0.001**(necrosis)
*Haemeophilus* spp.	0.474	0.594	0.994
*Klebsiella* spp.	**< 0.001** (necrosis)	**< 0.001** (tumor)	0.293
*Prevotella* spp.	0.794	**< 0.001** (abscess)	**< 0.001** (necrosis)
*Pseudomonas* spp.	0.072	**< 0.001** (tumor)	**< 0.001** (tumor)
*Staphylococcus* spp.			
All	**< 0.001** (abscess)	0.941	**< 0.001** (tumor)
*S. aureus*	0.090	**0.018** (tumor)	0.559
*Streptococcus* spp.			
All	**< 0.001** (abscess)	**< 0.001** (abscess)	**0.054** (necrosis)
β-hemolytic	0.855	**< 0.001** (tumor)	**0.002** (tumor)
*Veillonella* spp.	**0.025** (necrosis)	**0.022** (abscess)	**< 0.001** (necrosis)

**Fig. 4:**
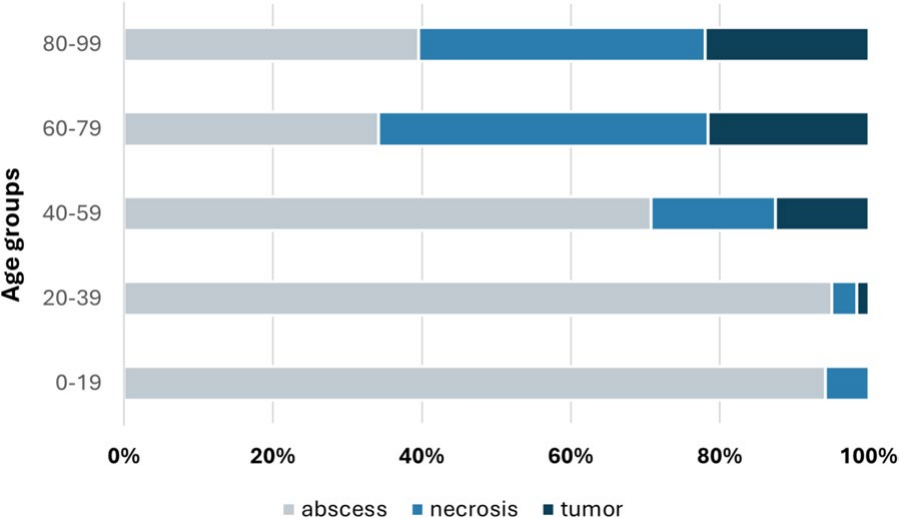
Distribution of diseases among the different age groups

The disease distributions across the different age groups are depicted in Fig. 4. Above the age of 40 years, an increase in the number of patients treated for necrosis or cancer was observed. In the 60–79-year-old age group, almost half of the patients were treated for necrosis (44.2%). In both the latter and the 80–99-year-old age groups, approximately one-fifth of the patients were diagnosed with oral cancer (21.6% and 22.0%, respectively).

### Antimicrobial resistance

**Fig. 5:**
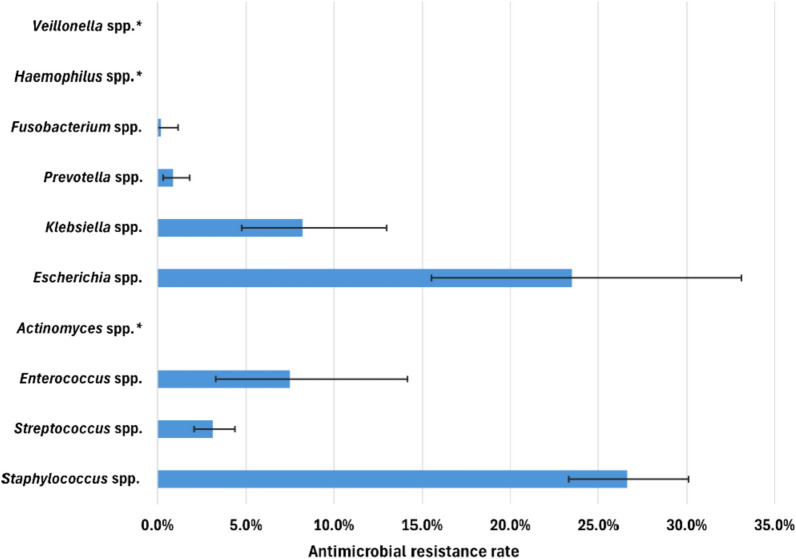
Antimicrobial resistance rates to amoxicillin-clavulanic acid. * No isolates were resistant

**Fig. 6:**
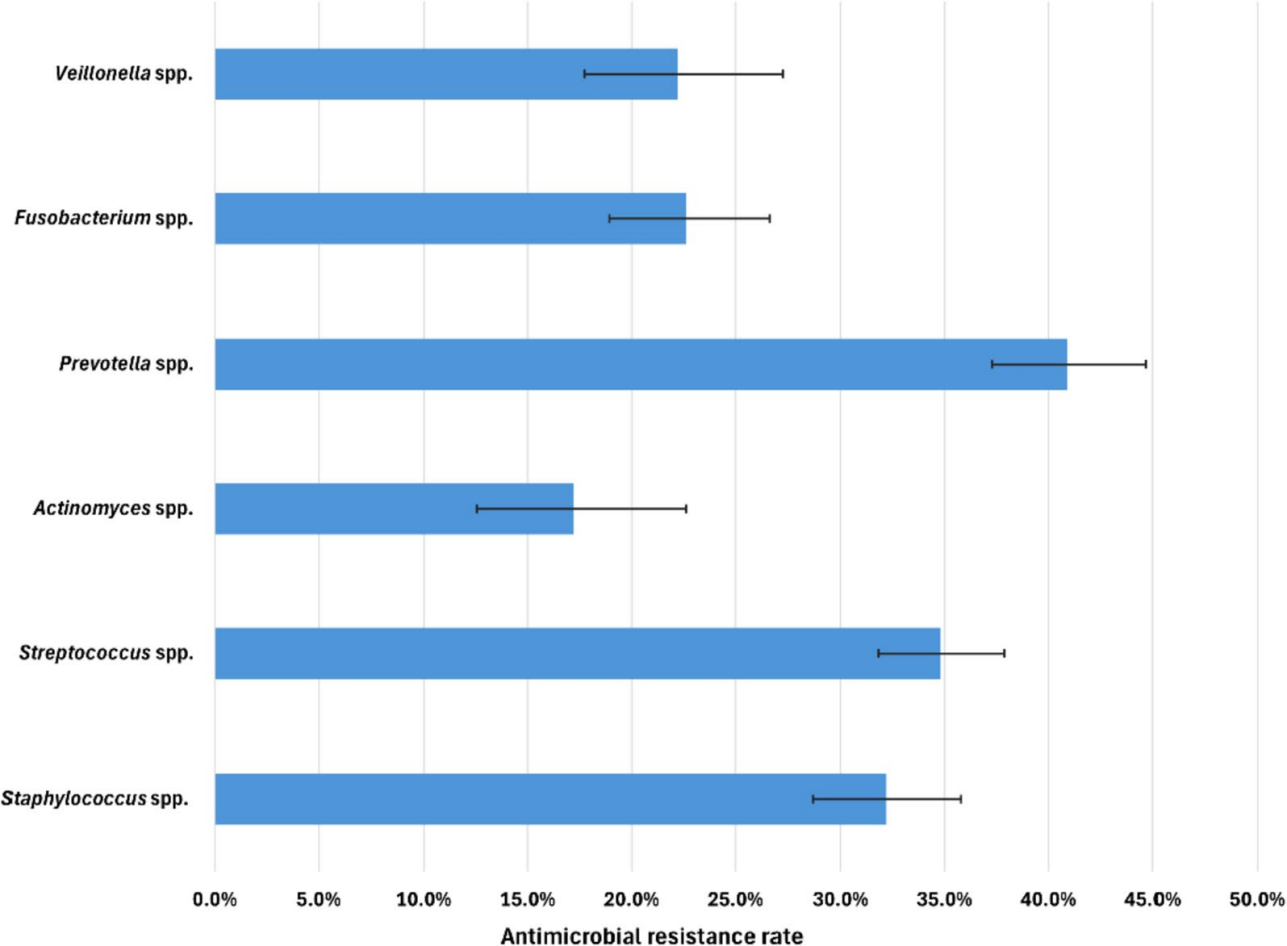
Antimicrobial resistance rates to clindamycin

Resistance rates to amoxicillin-clavulanic acid (AMC) and clindamycin (Figs. 5 and 6) have been highlighted, as these agents were prescribed as first- and second-line antibiotics in dental and maxillofacial settings in Hungary until recently, when the application of the current guidelines [25, 26] started. *Staphylococcus* spp. and *Escherichia* spp. had the highest resistance rates, with 26.6% (95% CI [23.3–30.1%]) and 23.5% (95% CI [15.5–33.1%]), respectively. *Streptococcus* spp. exhibited a 3.1% (95% CI [2.1–4.4%]) resistance rate. Resistance of *Enterobacter* spp. was not indicated, as only *Proteus mirabilis* was susceptible to AMC among those bacteria that were included in this group.

Substantial resistance rates to clindamycin were observed for *Prevotella* spp., *Streptococcus* spp. and *Staphylococcus* spp. at 40.9% (95% CI [37.3–44.7%]), 34.8% (95% CI [31.8–37.9%]) and 32.3% (95% CI [28.8–35.9%]), respectively. In addition, high resistance rates to clindamycin were observed for *Fusobacterium* spp., with a 22.6% resistance rate (95% CI [18.9–26.7%]), *Veillonella* spp., with a 22.2% resistance rate (95% CI [17.7–27.2%]), and *Actinomyces* spp., with a 17.2% resistance rate (95% CI [12.6–22.6%]).

**Fig. 7:**
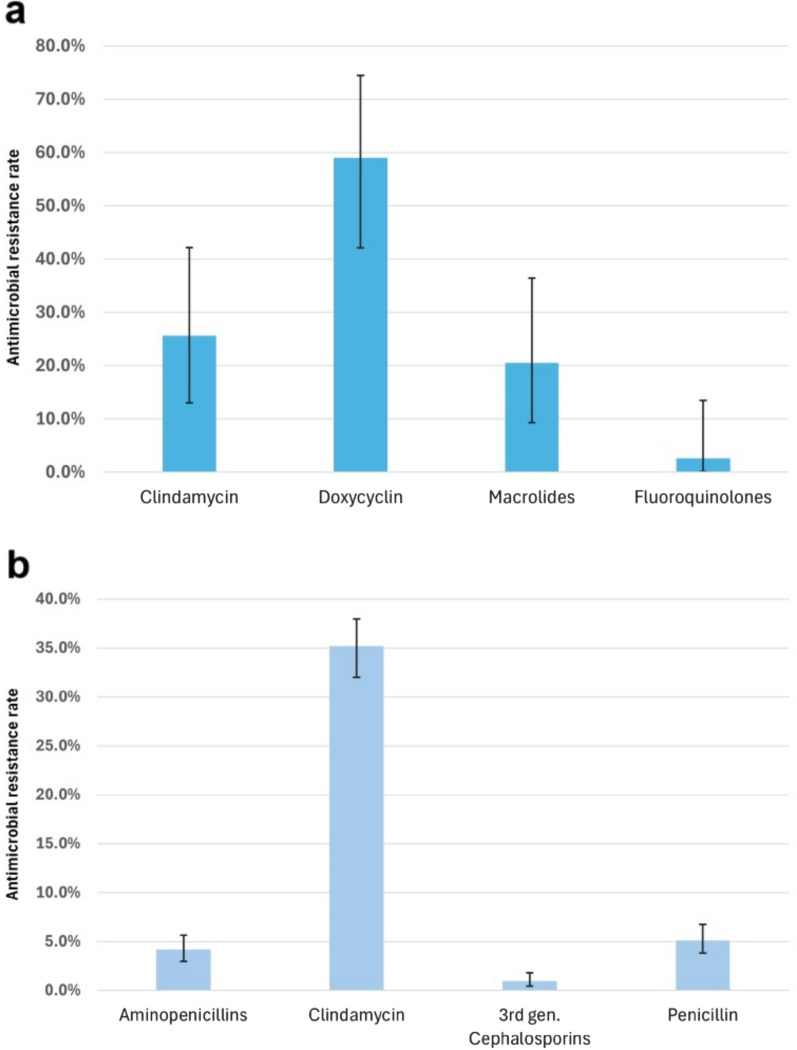
Antimicrobial resistance rates of streptococci. **a** beta-hemolytic streptococci. **b** other streptococci (viridans group)

The resistance rates of beta-hemolytic and other *Streptococcus* strains to the applicable antibiotics are depicted in Fig. 7. Compared with beta-hemolytic streptococci*,* viridans streptococci had an almost ten percent greater resistance rate to clindamycin. The latter group had substantial resistance to doxycycline (59%, 95% CI [42.1–74.4%]), and the resistance rate to macrolides was also not negligible (20.5%, 95% CI [9.3–36.5%]). The viridans group of streptococci presented relatively low resistance to beta-lactam antibiotics. Beta-hemolytic streptococci have 100% susceptibility to penicillin derivatives; hence, these were not portrayed here.

**Fig. 8:**
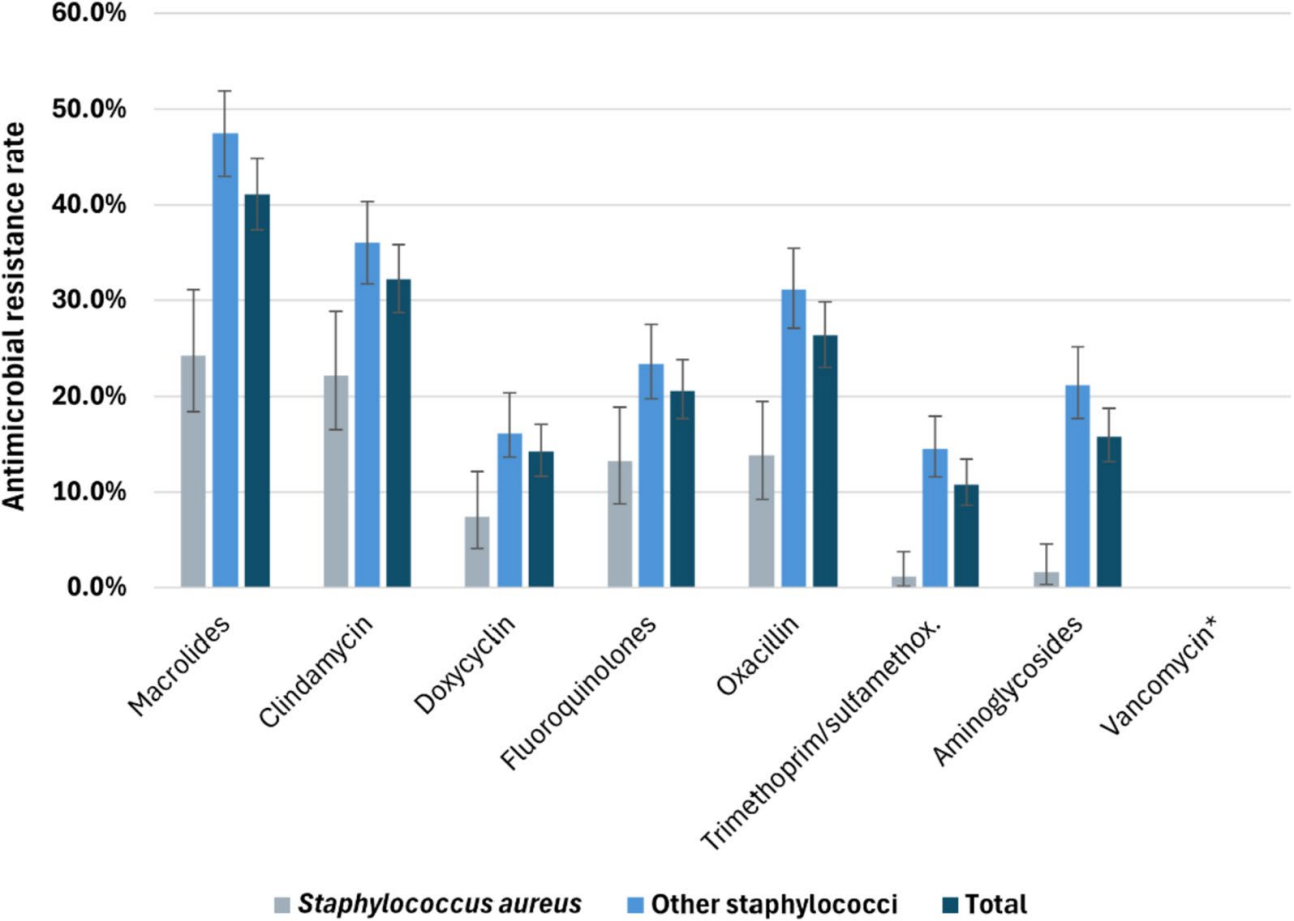
Antimicrobial resistance rates of Staphylococcus spp. * No isolates were resistant

The resistance rates of the *Staphylococcus* spp. are shown in Fig. 8. For all the species, the resistance rates were greater than 10% against all the antibiotic agents except for vancomycin, to which none of the isolates were resistant. Notably, *S. aureus* had distinctly lower resistance rates than the other staphylococci did, which consisted mainly of *S. epidermidis*. The percentage of methicillin-resistant *S. aureus* (MRSA) was 13.8% (95% CI [9.2–19.5%]) on average over the 6 years but fluctuated between 20% (2021) and 6.9% (2022) throughout the years. The percentage of methicillin-resistant *S. epidermidis* (MRSE) was 29.7% (95% CI [25.6–34.4%]).

**Fig. 9:**
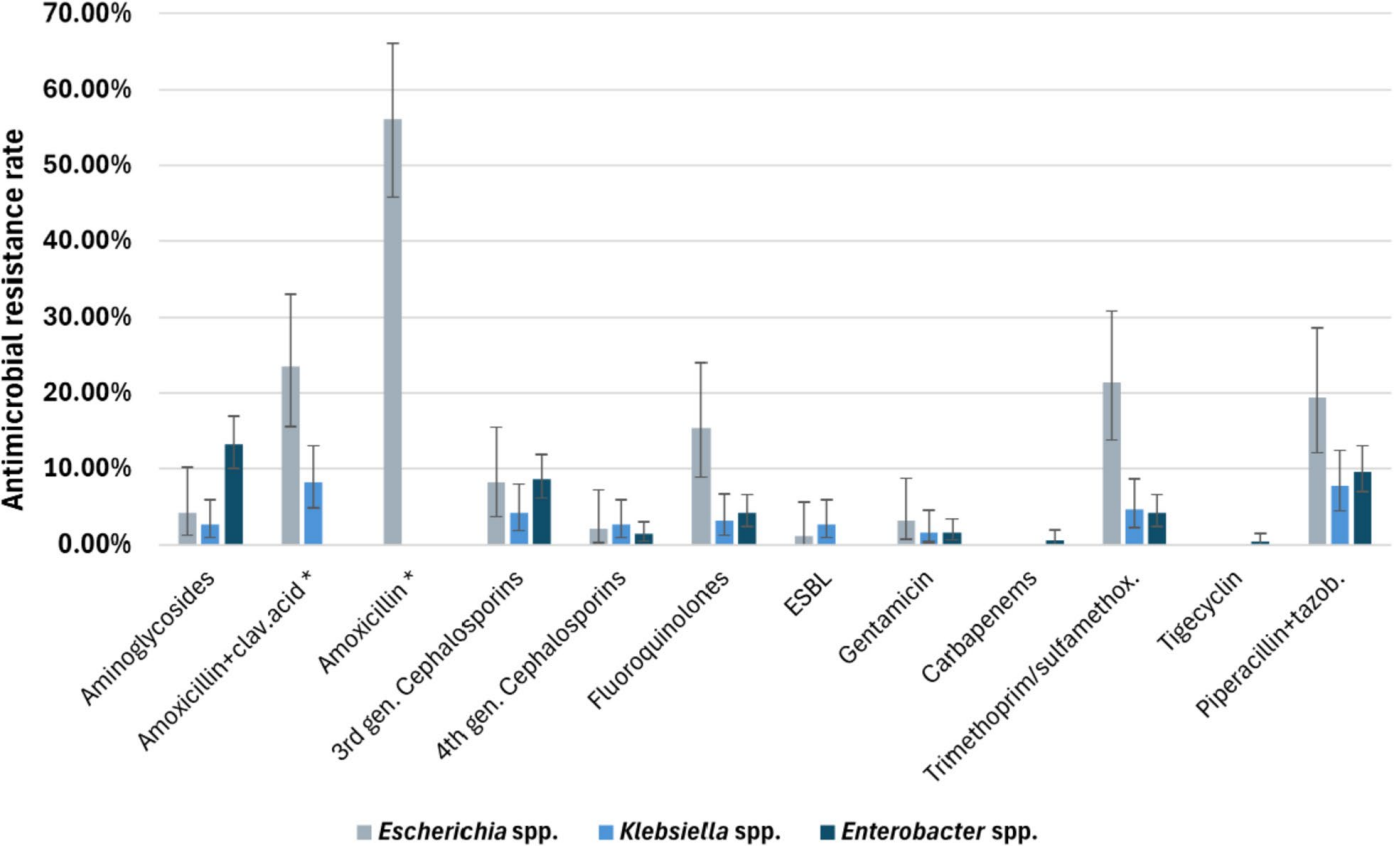
Antimicrobial resistance rates of Enterobacterales*.* * Bacteria with intrinsic resistance were not tested

The resistance rates of the members of the *Enterobacterales* order are shown in Fig. 9. As outlined above, *Escherichia* spp. demonstrated high resistance rates to penicillin derivatives and also fairly high resistance rates to trimethoprim-sulfamethoxazole and fluoroquinolones, with rates of 21.4% (95% CI [13.8–30.9%]) and 15.3% (95% CI [8.8–24.0%]), respectively. *Enterobacter* spp. had a 13.2% (95% CI [10–16.9%]) resistance rate to aminoglycosides. The percentages of extended-spectrum beta-lactamase (ESBL)-producing *Escherichia* spp. and *Klebsiella* spp. were 1.0% (95% CI [0.02–5.6%]) and 2.6% (95% CI [0.8–5.9%]), respectively.

*Enterococcus* spp. had a noteworthy 25.2% (95% CI [17.3–34.6%]) resistance rate to high-level gentamicin, and the percentage of vancomycin-resistant enterococci (VRE) was 2.8% (95% CI [0.6–8.0%]). The percentage of multidrug-resistant* P. aeruginosa* (MDRPA) was 1.1% (95% CI [0.03–6.0%]). Overall, the resistance rates fluctuated over the 6 years, but no specific increase or decrease was identified.

## Discussion

In this retrospective analysis, the spectrum of the isolated pathogenic bacteria and their AMRs were evaluated. The most frequently isolated bacteria were *Streptococcus*, *Prevotella*, *Staphylococcus* and *Fusobacterium* spp. Notably, *Enterobacter* spp. were the fifth most commonly isolated bacteria. Differences in the microbial composition of the three patient groups (‘abscess’, ‘necrosis’ and ‘tumor’) were also assessed. In conclusion, patients with malignant tumors had significantly greater percentages of *Enterobacter* spp., *Enterococcus* spp., *Pseudomonas* spp. and beta-hemolytic streptococci than did the other two patient groups. Patients in the ‘abscess’ group had significantly lower percentages of *Enterobacter* spp., *Enterococcus* spp., *Escherichia* spp. and *Klebsiella* spp. than patients in the ‘necrosis’ and ‘tumor’ groups did. Compared with patients in the other groups, patients in the necrosis group had significantly greater percentages of *Actinomyces* spp. Among our findings regarding AMR, the particularly high resistance rates to clindamycin among *Prevotella*, *Streptococcus* and *Staphylococcus* spp. should be highlighted.

### Comparison of the pathogen spectrum and AMR pattern to available worldwide data

Although the antimicrobial spectrum and AMR among patients with maxillofacial infections have been studied worldwide, the methodologies of these studies were unfortunately not consistent in terms of susceptibility testing or evaluation of the pathogen spectrum. Most of the studies were performed with a smaller population, and the bacterial spectrum was often divided into aerobes and anaerobes or gram-positive and gram-negative bacteria rather than being evaluated as a whole. Moreover, in some cases, resistance rates were reported for the entirety of the bacteria rather than individually for each genus, and the same guidelines were not adopted for the evaluation of susceptibility throughout the studies, making comparisons difficult.

In general, the bacteria most frequently isolated worldwide are *Streptococcus* spp., followed by *Prevotella* and *Staphylococcus* spp*.* [27–37]. This finding is in accordance with our results, although the spectrum of pathogens in our population exhibited greater diversity; thus, the percentage of streptococci was not as high. The majority of these studies focused only on odontogenic infections, which may explain the difference in diversity compared with our study. However, the pathogen spectrum in the ‘abscess’ group was also fairly different in composition compared with data from other countries, which could be expected owing to the changes in the microbiome in geographically distinct populations [38]. According to the literature, the microbes involved in SSIs in general are *S. aureus*, coagulase-negative staphylococci, *E. coli*, *E. faecalis*, and *P. aeruginosa* [39]. A study conducted in India specifically focused on SSIs in head and neck cancer patients revealed that *Klebsiella* spp., *Acinetobacter* spp., *E. coli*, *S. aureus* and *Enterococcus* spp. were the most common bacteria among the isolates from these patients [40], which is only somewhat similar to our results.

With respect to AMR, we compared our results only with studies in which the AMR of each genus was evaluated separately. Notably, even the European studies were not completely consistent with the EUCAST guidelines that our laboratory uses [24]. With respect to these limitations, we could draw relevant conclusions for only a few antibiotics. The resistance rate to clindamycin ranged from 13.7% [28] to 46% [35] in the viridans streptococci group. Resistance rates estimated in different parts of the world, specifically in the U.S. [29, 33], Germany [30] and Iraq [36], were consistent with our rates (35.2%). An outstanding result in our study was the 40.9% resistance rate of *Prevotella* spp. to clindamycin, which is substantially higher than the data in the literature, where the highest AMR rate was reported to be 22.5% [30, 31, 35, 36]. Notably, in almost every country, the resistance rate of *S. aureus* to vancomycin was 0%, including in our study, except for Iraq [36], where a remarkable 30.4% resistance rate was estimated. In terms of structure and scope, the study most similar to ours was conducted by Meinen et al. in Germany, in which they compared the data acquired from hospitals to those acquired from dental practices [27]; however, it should be noted that they reported only resistance rates to *S. aureus*, *Streptococcus* and *Klebsiella* spp. Compared with their results from hospital settings, we observed substantially higher resistance rates to clindamycin among both *Streptococcus* spp. (32.2% vs. 19.4%) and *S. aureus* (22.2% vs. 17%) and slightly higher percentages of MRSA (13.8% vs. 12.0%). However, in terms of *Klebsiella* spp., our outcomes were more similar to the data from dental practices, with a 4.1% resistance rate to third-generation cephalosporines, a 3.1% resistance rate to fluoroquinolones and no resistance to carbapenems.

### The role of *S. epidermidis* in oral biofilm formation and its relationship with systemic diseases

One of the main virulence factors of *S. epidermidis* is biofilm formation, which is particularly associated with the colonization of surgical wounds or medical devices such as implants. Although *S. epidermidis* is mostly regarded as a commensal microorganism of human skin and mucosal surfaces, including the oral cavity, it can also emerge as an opportunistic pathogen, especially in immunocompromised patients or those undergoing surgery. There is evidence suggesting that *S. epidermidis* may function as a virulence gene reservoir that can potentially increase the pathogenic potential of *S. aureus* through horizontal gene transfer. *S. epidermidis* has emerged as one of the leading causes of nosocomial infections; however, it can also play a favorable role in suppressing the outgrowth of aggressive pathogens, primarily *S. aureus*. It can spread from the oral cavity to other body sites, potentially contributing to systemic infections such as infective endocarditis, prosthetic joint infections, and device-related osteomyelitis [11, 12, 41–43]. In addition to the inadequate oral hygiene encountered in the Hungarian population [23], other factors could also contribute to biofilm formation, which can also explain the relatively high percentage of *S. epidermidis* in our study compared with other studies [27]. These factors include titanium implants or osteosynthesis plates inserted intraorally, as well as surgical interventions breaking through the skin (e.g., extraoral incisions or neck dissections).

### The relevance of oral pathogens in carcinogenesis

It has been estimated that 15–20% of human tumors can be induced by infections. The aim of some of the studies on this topic was to identify early diagnostic markers within the microbiome that could be helpful in the early detection of cancer. On the other hand, studies on the microbiome of cancer patients also revealed that patients undergoing chemoradiotherapy may have alterations in their oral microbiota induced by treatment, which can lead to other systemic health problems through the emergence of potential pathogens [10, 15, 18, 44].

There has been increasing concern regarding the possible role of *Fusobacterium* spp. in promoting carcinogenesis [18, 45–49]. Yosat et al. [50] reported that these bacteria were metabolically hyperactive in the oral microbiome of patients with OSCC. According to their research, *Fusobacteria* were significantly more active in tumor sites than in tumor-adjacent sites, and these authors also demonstrated the greatest upregulation of the expression of putative virulence factors. However, our results were not consistent with these findings, as patients with malignant tumors presented significantly lower percentages of *Fusobacterium* spp. than did those in the ‘abscess’ or ‘necrosis’ groups, although importantly, molecular biology techniques were utilized in the aforementioned studies. Cai et al. [51] reported an enrichment of *Fusobacterium nucleatum* in the tumor microenvironment compared with healthy sites, but interestingly, *F. nucleatum* enrichment was significantly associated with nonsmokers, nondrinkers and a better survival rate, suggesting that *F. nucleatum* enrichment can be an indicator of more favorable outcomes in oral cancer patients. Our results regarding the lower percentages of *Fusobacterium* spp. might support this theory, as Hungary has the highest mortality and morbidity rates of oral cancer patients in Europe and a considerably low overall 5-year survival rate, with smoking and alcohol consumption reported to be the most important risk factors [52, 53]. This assumption was further supported by the findings of Eun et al. [22], who reported that *Prevotella* spp. were enriched in the saliva of patients with lymph node metastasis, whereas *Fusobacterium* spp. were dominant in patients without metastasis. Other pathogens that are strongly associated with OSCC are *P. aeruginosa* [48]*, Porphyromonas gingivalis* and *Prevotella intermedia*, but other bacterial genera, such as *Actinomyces,* members of the *Enterobacteriaceae* family*, Haemophilus, Streptococcus* and *Veillonella*, are also associated with oral cancer [54]. Although most studies have focused on either tumor surfaces or intratumoral tissue, many have reported overlapping bacterial genera, indicating a common core microbiota associated with OSCC. Gopinath et al. [55] and Nagy et al. [16] both found that tumor surfaces were enriched with genera such as *Fusobacterium, Porphyromonas*, and members of the *Enterobacteriaceae* family, whereas Gopinath noted that *Prevotella* and *Treponema* were more abundant within tumor tissue. Additionally, Nagy et al. reported elevated levels of *Veillonella*, *Actinomyces*, *Clostridium*, *Haemophilus*, and *Streptococcus* spp. in biofilms on tumor surfaces. Furthermore, Hooper et al. [17] found that the intratumoral microbiota was predominantly saccharolytic and aciduric, including Proteobacteria and genera such as *Fusobacterium, Streptococcus, Prevotella*, and *Veillonella*, which they suggested may reflect selective bacterial growth within carcinoma tissue. However, the presence of similar taxa on tumor surfaces raises the possibility that such bacteria may not be exclusively selected by the intratumoral environment and could instead result from bacterial migration from the surface. Our results are only partially in line with these findings, since these pathogens were not specifically increased in cancer patients; in fact, most patients in the cancer group had significantly lower percentages of these species than did patients in the abscess or necrosis groups, and only *Enterobacter* spp. and beta-hemolytic streptococci were present in significantly higher percentages in this particular patient group. We must note that the samples taken from these patients were not exclusively taken intraorally but also from neck surgical sites, but these sites are often connected to the oral wound, thereby resulting in infection [56]. Panghal et al*.* reported similar results to ours in their microbiological analysis of oral cancer patients [57], where the most prevalent bacteria were *Staphylococcus* spp., *E. coli*, *K. pneumoniae* and *Proteus* spp. Our results were also consistent with the outcomes of the study conducted by Jobbins et al. [58], who evaluated the oral pathogens of patients with advanced malignant diseases and reported that terminally ill patients presented an increase in coliform bacteria. In addition to the patients treated for oral cancer, the majority of the patients in the ‘necrosis’ group could also be considered terminally ill patients in our study, as the common underlying diseases of patients treated for osteonecrosis were usually breast cancer, prostate cancer or multiple myeloma. These patients often receive antiresorptive drugs, which might also cause a shift in the microbiota of the mouth, as the reduced resilience of the bone can lead to opportunistic infections. Various studies have focused on the role of periodontopathogens (e.g., *Fusobacterium, Prevotella,* and *Porphyromonas* spp.) and *Actinomyces* spp., but in a thorough evaluation of the results of these studies, we observed a considerable percentage of *Proteobacteria* in the isolates of these patients [59–61]. These findings are in accordance with our results, as the patients in the ‘necrosis’ group presented increased percentages of *Actinomyces* and *Prevotella* spp., and similar to the patients in the ‘tumor’ group, they also presented substantial percentages of *Enterobacter, Enterococcus, Escherichia* and *Klebsiella* spp. It has been previously suggested that coliform bacteria could be used as markers for underlying diseases, as these types of bacteria are practically absent from the oral cavity of healthy patients [13, 58]. A study by Karpinets et al. [62] revealed that the intratumoral microbiome of adenoid cystic carcinoma patients featured gut-like bacteria, with low diversity and colonization by *Proteobacteria* and other gut microbes, such as *Enterococcus* spp., which were negatively associated with patient survival compared with the bacteria typically found in the oral cavity. There is growing evidence suggesting that the oral microbiota may play a role in the development of diseases of the gastrointestinal system, such as colorectal cancer [63], but the reverse interaction between the oral cavity and the gut microbiome might be due to the lower resistance of the intratumoral microbiome to colonization by gut bacteria. Although we are aware that the generally stable oral microbiota of healthy individuals can be disrupted by various local and systemic diseases, the exact role of bacteria in the pathomechanisms of the aforementioned diseases is still unclear, despite the efforts of numerous studies. Furthermore, the oral microbiota can potentially influence the morbidity of patients who are already compromised, especially those with advanced disease, but further investigation is needed.

This study represents the first comprehensive microbiological evaluation of maxillofacial infections in Hungary and, to our knowledge, is the first to compare the pathogen spectrum between oral cancer patients and those treated for nonmalignant lesions, specifically abscesses and necrosis. Our results revealed significantly greater percentages of Enterobacterales among the clinical isolates of patients with oral cancer than among the other patient groups. The high abundance of Enterobacterales in oral cancer patients, coupled with the intrinsic resistance of many bacteria in this order to amoxicillin with clavulanic acid—the commonly used first-line empirical antibiotic therapy—highlights the need for targeted microbial surveillance, early detection, and personalized treatment strategies to prevent severe infections and complications during cancer treatment and recovery. Future studies are needed to determine whether Enterobacterales are present in intratumoral tissues, contributing to the development of SSIs, or if they are introduced as nosocomial infections during hospital care. Another key finding of our investigation was the high rate of clindamycin resistance, particularly among *Prevotella*, *Streptococcus* and *Staphylococcus* spp. The high resistance rates to clindamycin in head and neck infections highlight the need to reconsider its empirical use and promote a more personalized, susceptibility-based approach, emphasizing the importance of minimizing unnecessary antibiotic use to prevent AMR and improve patient outcomes. However, several limitations should be considered when interpreting these findings. Bacterial identification was based solely on conventional culturing techniques without the use of molecular diagnostic methods. While this may have limited the detection of uncultivable organisms, it is important to note that culture-based methods remain essential for phenotypic antimicrobial resistance profiling, which was a primary objective of the study. Variability in sample collection methods—specifically between intraoral and extraoral approaches—and the fact that the isolates from cancer patients in this study were often not obtained directly from intratumoral tissue may have influenced the microbiological findings. Additionally, detailed clinical background information was not consistently available for all patients, which prevented the inclusion of these data and limited the ability to explore associations between clinical factors, microbiological profiles and AMR rates. Future studies incorporating standardized sampling methods and molecular diagnostics, alongside complete clinical data, would provide a more nuanced understanding of pathogen diversity and resistance dynamics in head and neck infections.

## Conclusion

Understanding the most common oral pathogens and AMR patterns in different geographical regions is essential for developing appropriate antibiotic stewardship programs. Our study revealed a significant abundance of Enterobacterales in patients with malignancies. Particularly high resistance rates were observed for clindamycin among *Prevotella* spp., coagulase-negative staphylococci and viridans group streptococci*.* Given that these bacteria are prevalent in head and neck infections, the routine use of clindamycin as a second-line antibiotic for patients with penicillin allergies should be reassessed, and this reconsideration should be reflected in updated treatment guidelines developed through regional antibiotic stewardship programs. The application of uniform guidelines regarding antimicrobial susceptibility would be beneficial for future investigations. The microbiota might be involved in the pathogenesis of OSCC through the induction of inflammatory responses, and to clarify the exact pathways of this mechanism, studies have focused mostly on periodontopathogens such as *Fusobacterium*, *Porphyromonas* and *Prevotella* spp. We aim to investigate the potential involvement of intratumoral bacteria—particularly members of the Enterobacterales—in the pathogenesis of SSIs and in the carcinogenesis of oral malignancies in future studies, as their exact role in these processes remains to be determined. Additionally, we plan to investigate the AMR rate in different patient groups and its correlation with other influencing factors using a more comprehensive dataset.

## Supplementary Information

Additional file 1.

## Data Availability

The data that support the findings of this study are not openly available due to reasons of sensitivity and are available from the corresponding author upon reasonable request.

## Abbreviations

AMR: Antimicrobial resistance rate
AMC: Amoxicillin-clavulanic acid
CDC: Centers for Disease Control and Prevention
CI: Confidence interval
DLM: Department of Laboratory Medicine
ESBL: Extended-spectrum beta-lactamase
EUCAST: European Committee on Antimicrobial Susceptibility Testing
MALDI-TOF MS: Matrix-assisted laser desorption ionization–time of flight mass spectrometry
MDRPA: Multidrug-resistant* Pseudomonas aeruginosa*
MRSA: Methicillin-resistant *Staphylococcus aureus*
MRSE: Methicillin-resistant *Staphylococcus epidermidis*
OSCC: Oral squamous cell carcinoma
SSI: Surgical site infection
VRE: Vancomycin-resistant enterococci
WHO: World Health Organization
