# Supplementary material for: Enterobacterales abundance in oral cancer patients and elevated clindamycin resistance rates in head and neck infections at a Hungarian Tertiary Hospital
Source: Ann Clin Microbiol Antimicrob. 2025 May 29;24:33. doi: 10.1186/s12941-025-00802-x (PMC12124057; doi:10.1186/s12941-025-00802-x)
Supplement: Supplementary file 1 — Additional file 1. [file 12941_2025_802_MOESM1_ESM.docx]

**Additional file**

**Table 1.**Percentage distribution of bacteria identified from infections of the head and neck region.
*Percentages of S. aureus among all staphylococci **Percentages of beta-hemolytic streptococci among all streptococci

|  | | **n** | **%** |
| --- | --- | --- | --- |
| ***Actinomyces* spp.** | | 233 | 4.49% |
| ***Enterobacter* spp.** | | 394 | 7.60% |
| ***Enterococcus* spp.** | | 107 | 2.06% |
| ***Escherichia* spp.** | | 98 | 1.89% |
| ***Fusobacterium* spp.** | | 473 | 9.12% |
| ***Haemophilus* spp.** | | 62 | 1.20% |
| ***Klebsiella* spp.** | | 195 | 3.76% |
| ***Prevotella* spp.** | | 701 | 13.52% |
| ***Pseudomonas* spp.** | | 91 | 1.76% |
| ***Staphylococcus* spp.** | **all** | 495 | 13.19% |
|  | ***S. aureus*** | 189 | 3.65%/*27.63%** |
| ***Streptococcus* spp.** | **all** | 935 | 18.78% |
|  | **β - hemolytic** | 39 | 0.75%/*4.00%*** |
| ***Veillonella* spp.** | | 315 | 6.08% |
| **Others** | | 858 | 16.55% |
| **Total** | | 5185 | |

**Figure 1.**Percentages of bacteria in the different patient groups before the exclusion of all recurring isolates


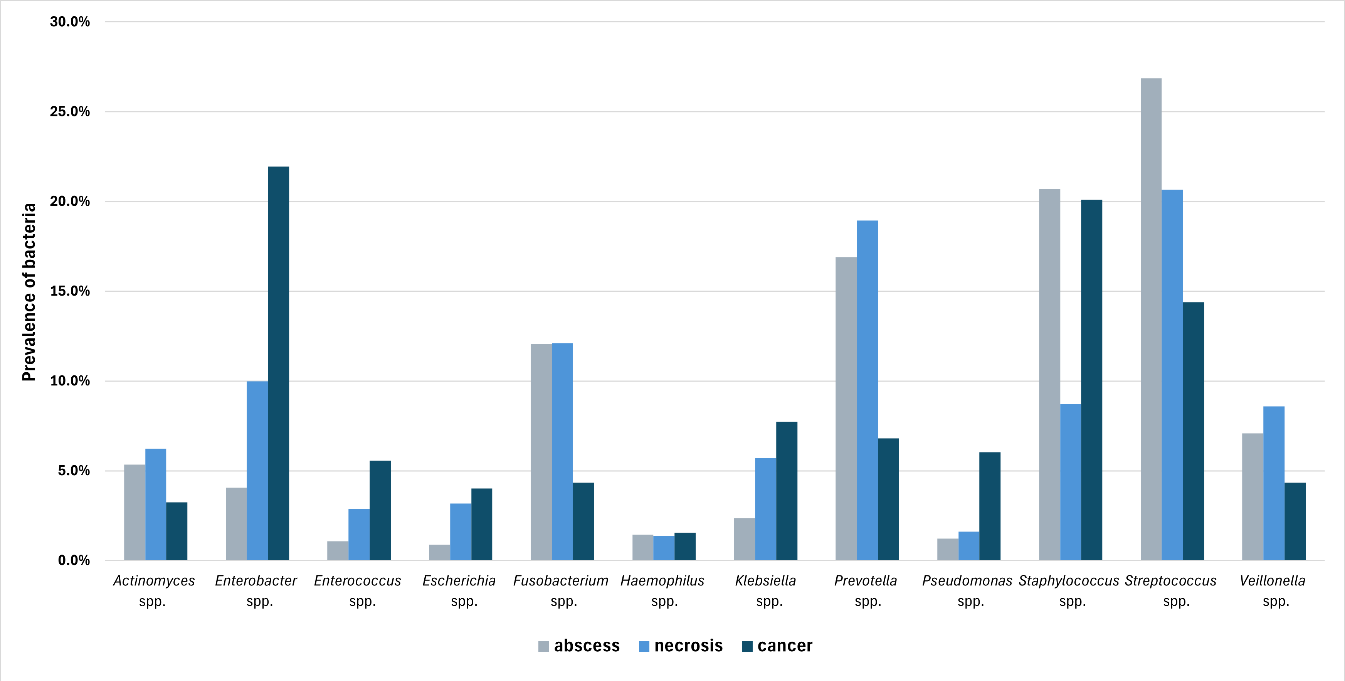


**Table 2.**

Percentages of bacteria after excluding all recurring isolates for Pearson’s chi-square test
*Percentages of S. aureus among all staphylococci
**Percentages of beta-hemolytic streptococci among all streptococci

|  | | **Abscess** | | **Necrosis** | | **Tumor** | | **All** | |
| --- | --- | --- | --- | --- | --- | --- | --- | --- | --- |
|  |  | **n** | **%** | **n** | **%** | **n** | **%** | **n** | **%** |
| ***Actinomyces* spp.** | | 96 | 5.6% | 92 | 7.6% | 17 | 3.4% | 205 | 5.9% |
| ***Enterobacter* spp.** | | 74 | 4.3% | 117 | 9.6% | 93 | 18.3% | 284 | 8.2% |
| ***Enterococcus* spp.** | | 20 | 1.2% | 40 | 3.3% | 31 | 6.1% | 91 | 2.6% |
| ***Escherichia* spp.** | | 17 | 1.0% | 44 | 3.6% | 23 | 4.5% | 84 | 2.4% |
| ***Fusobacterium* spp.** | | 224 | 13.0% | 158 | 13.0% | 26 | 5.1% | 408 | 11.8% |
| ***Haemophilus* spp.** | | 28 | 1.6% | 24 | 2.0% | 10 | 2.0% | 62 | 1.8% |
| ***Klebsiella* spp.** | | 45 | 2.6% | 83 | 6.8% | 42 | 8.3% | 170 | 4.9% |
| ***Prevotella* spp.** | | 247 | 14.3% | 178 | 14.6% | 33 | 6.5% | 458 | 13.3% |
| ***Pseudomonas* spp.** | | 20 | 1.2% | 24 | 2.0% | 30 | 5.9% | 74 | 2.1% |
| ***Staphylococcus* spp.** | **all** | 370 | 21.4% | 120 | 9.9% | 108 | 21.3% | 598 | 17.3% |
|  | ***S. aureus*** | 94 | 5.4%/  *25.4%** | 40 | 3.3%/  *33.3%** | 40 | 7.9%/  *37.0%** | 174 | 5.0%/  *29.1%** |
| ***Streptococcus* spp.** | **all** | 467 | 27.0% | 224 | 18.4% | 74 | 14.6% | 765 | 22.2% |
|  | **β - hemolytic** | 18 | 1.0%/  *3.85%*** | 8 | 0.7%/  *3.6%*** | 10 | 2.0%/  *13.5%*** | 36 | 1.0%/  *4.7%*** |
| ***Veillonella* spp.** | | 120 | 6.9% | 112 | 9.2% | 21 | 4.1% | 253 | 7.3% |
| **Total** | | 1728 | | 1216 | | 508 | | 3452 | |
